# Screening and development of monoclonal antibodies for identification of ferret T follicular helper cells

**DOI:** 10.1038/s41598-021-81389-z

**Published:** 2021-01-21

**Authors:** Wenbo Jiang, Julius Wong, Hyon-Xhi Tan, Hannah G. Kelly, Paul G. Whitney, Ian Barr, Daniel S. Layton, Stephen J. Kent, Adam K. Wheatley, Jennifer A. Juno

**Affiliations:** 1 Department of Microbiology and Immunology, Peter Doherty Institute for Infection and Immunity, University of Melbourne, Melbourne, VIC Australia; 2 WHO Collaborating Centre for Reference and Research On Influenza, Peter Doherty Institute for Infection and Immunity, University of Melbourne, Melbourne, VIC Australia; 3 CSIRO Health and Biosecurity, Australian Animal Health Laboratories, Geelong, VIC Australia; 4 Melbourne Sexual Health Clinic and Infectious Diseases Department, Alfred Hospital, Monash University Central Clinical School, Carlton, VIC Australia; 5 ARC Centre for Excellence in Convergent Bio-Nano Science and Technology, University of Melbourne, Melbourne, Australia

**Keywords:** Follicular T-helper cells, Lymph node

## Abstract

The ferret is a key animal model for investigating the pathogenicity and transmissibility of important human viruses, and for the pre‐clinical assessment of vaccines. However, relatively little is known about the ferret immune system, due in part to a paucity of ferret‐reactive reagents. In particular, T follicular helper (Tfh) cells are critical in the generation of effective humoral responses in humans, mice and other animal models but to date it has not been possible to identify Tfh in ferrets. Here, we describe the screening and development of ferret-reactive BCL6, CXCR5 and PD-1 monoclonal antibodies. We found two commercial anti-BCL6 antibodies (clone K112-91 and clone IG191E/A8) had cross-reactivity with lymph node cells from influenza-infected ferrets. We next developed two murine monoclonal antibodies against ferret CXCR5 (clone feX5-C05) and PD-1 (clone fePD-CL1) using a single B cell PCR-based method. We were able to clearly identify Tfh cells in lymph nodes from influenza infected ferrets using these antibodies. The development of ferret Tfh marker antibodies and the identification of ferret Tfh cells will assist the evaluation of vaccine-induced Tfh responses in the ferret model and the design of novel vaccines against the infection of influenza and other viruses, including SARS-CoV2.

## Introduction

Ferrets (Mustela putorius furo) are a well-established animal model for influenza research and are widely used to investigate the pathogenesis and transmission of influenza viruses and pre‐clinically evaluate the efficacy of influenza vaccines^1,2^. In addition, ferrets serve as an animal model for the study of other viruses, including human respiratory syncytial virus (HRSV)^3^, human metapneumovirus (HMPV)^4^, Hendra virus (HeV)^5^, Nipah virus (NiV)^5^, different species of ebolavirus^6^ and severe acute respiratory syndrome coronavirus (SARS‐CoV)^7^. Martina et al. demonstrated that ferrets are susceptible to experimental infection by SARS‐CoV, and that the virus is efficiently transmitted to animals living with them^8^. It has now been shown that ferrets are similarly susceptible to the pandemic virus severe acute respiratory syndrome coronavirus 2 (SARS-CoV-2), and that the virus replicates efficiently in the upper respiratory tract of ferrets^9,10^. Ferrets are therefore a useful model in which to assess neutralizing antibody responses to viral challenge and to test novel vaccine candidates^11^.

Characterization of the ferret immune response following infection and/or vaccination is informative for developing vaccines and anti-viral therapies. However, a diverse range of ferret-specific immunological reagents are not currently available. One major gap is the lack of reagents to study T follicular helper (Tfh) cells, which are critical for the generation and maturation of the antibody response. Tfh cells are a subset of CD4 T cells that provide help with B cells for high-affinity antibody production in germinal centres (GC)^12–14^. These specialised cells are crucial for the formation of GC, affinity maturation and maintenance of B cell memory. BCL6 is the master transcription factor for Tfh differentiation. Distinguishing phenotypic markers of Tfh cells include the high expression of CXCR5, PD-1, and ICOS^12^. High CXCR5 expression facilitates Tfh cells migration to B cell follicles where its ligand, CXCL13 is produced abundantly by follicular stromal cells. PD-1 and ICOS are required to engage with their respective ligands, PD-L1/PDL-2 and ICOSL, which are expressed by GC B cells to support the development of Tfh cells^12^.

Here, we describe the screening and development of ferret Tfh marker monoclonal antibodies and identification of ferret Tfh cells using these antibodies. We first identified two commercial anti-human/mouse BCL6 antibodies which had cross-reactivity with ferret lymph node (LN) cells. We next developed mouse anti-ferret CXCR5 and PD-1 monoclonal antibodies using single cell PCR-based method. Finally, we detected Tfh cells in lymph nodes from influenza infected ferrets using these antibodies.

## Results

### Screening of commercial anti-human or mouse BCL6, CXCR5 and PD-1 antibodies for cross-reactivity with ferret lymph node cells

**Figure 1:**
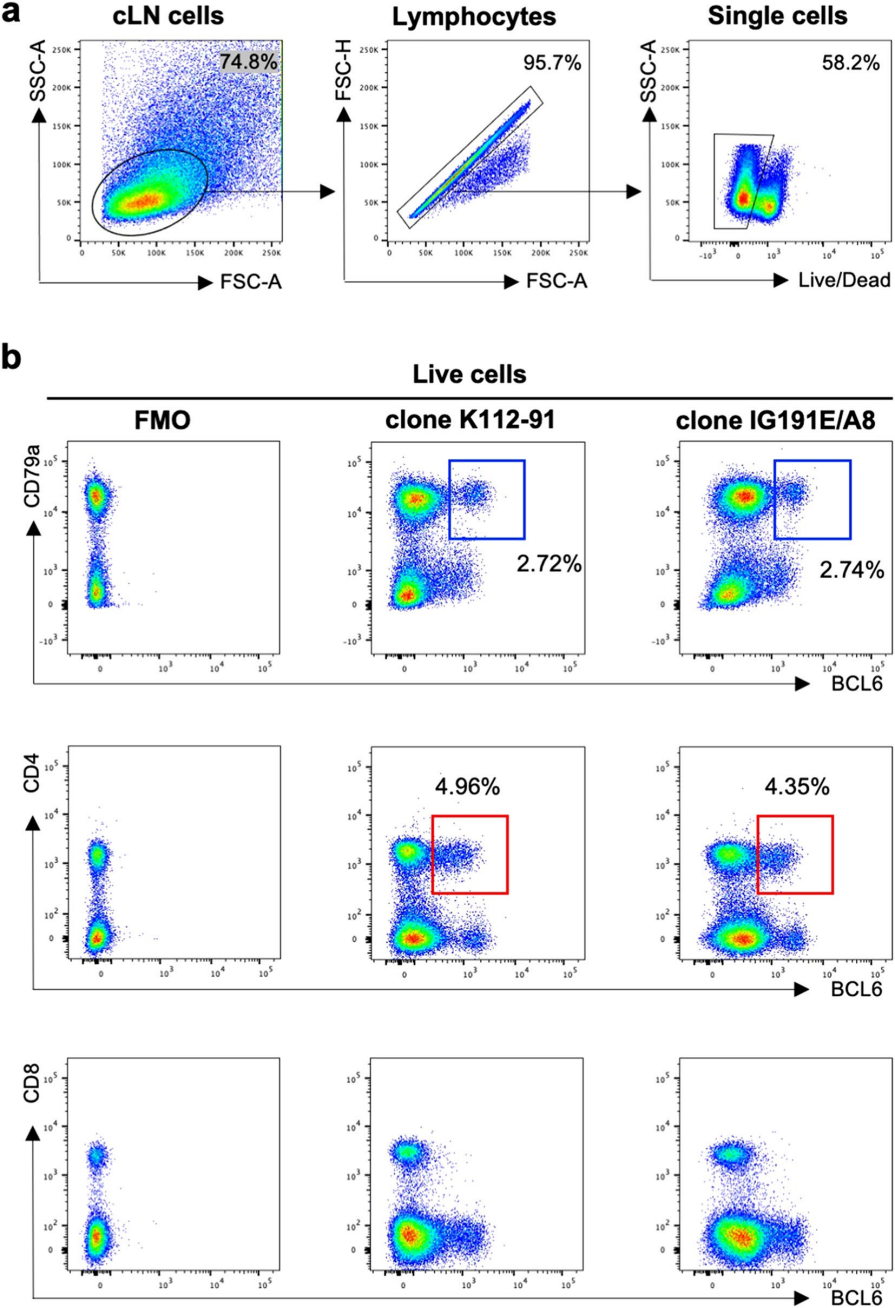
Cross-reactivity of commercial anti-human/mouse BCL6 antibodies with lymph node cells from influenza infected ferret. (**a**) Gating strategy to identify live lymphocytes in the ferret LN. Lymphocytes were identified by forward scatter area (FSC-A) and side-scatter area (SSC-A). Doublets were excluded by gating on single cells as determined by FSC-A versus FSC-H, and live cells were identified by viability dye exclusion. For each step, the parental population is indicated above the plot. (**b**) Representative plots of influenza-infected ferret lymph node cells stained with anti-BCL 6 (clone K112-91 and clone IG191E/A8), CD79a, CD4 and CD8 antibodies. The parental population is indicated above the plot. Blue box indicates GC B cells (BCL6+CD79a+) and red box indicates Tfh cells (BCL6+CD4+).

BCL6 expression is the canonical transcription factor that distinguishes Tfh cells from other CD4+ T cells in human and mouse^15–17^. However, high co-expression of CXCR5 and PD-1 serves as surrogate or confirmatory surface markers for the Tfh population in human and mouse lymphoid tissues^18^. Commercial anti-human/mouse BCL6 antibodies were screened for cross-reactivity against ferrets by staining LN cell suspensions recovered from influenza infected ferrets. The gating strategy to identify live lymphocytes in the ferret LN is shown in Fig. 1a. We found that clones K112-91 and IG191E/A8, originally developed for human BCL6^15,16^, showed cross-reactivity with ferret cLN cells (Fig. 1b). The BCL6+ B cell (CD79a+) population represents a putative GC B cell population, while BCL6+ CD4+ cells are likely to mark Tfh cells although additional markers are needed.

We next screened commercial antibodies raised against mouse or human CXCR5 (clones L138D7 and RF8B2) and PD-1 (clones 29F.1A12 and EH12.2H7) for ferret cross-reactivity. Unfortunately, all screened murine and human antibodies showed no cross-reactivity with ferret lymph node cells (data not shown) although they showed good reactivity with mouse or human lymph node cells. Thus, we initiated the generation of ferret CXCR5 and PD-1-specific monoclonal antibodies.

### Homology analysis of ferret, human and mouse BCL6, CXCR5 and PD-1

Comparison of amino acid homology of ferret, human and mouse BCL6, CXCR5 and PD-1 confirmed ferret BCL6 was a highly conserved, with high homology to both human BCL6 (95.47%) and mouse BCL6 (93.78%) (Table 1). In contrast, ferret CXCR5 had only moderate homology with human CXCR5 (84.22%) and mouse CXCR5 (87.43%) and ferret PD-1 had low homology with both human (67.24%) and mouse PD-1 (54.48%), consistent with the lack of ferret cross-reactivity of commercially available CXCR5 and PD-1-specific monoclonal antibodies.

**Table 1:** Antigen homology of ferret, human and mouse BCL6, CXCR5 and PD-1.

Antigen	Antigen homology (%)		
	Ferret vs human	Ferret vs mouse	Human vs Mouse
BCL6	95.47	93.78	94.63
CXCR5	84.22	87.43	83.16
PD-1	67.24	54.48	59.31

### Generation of mouse anti-ferret CXCR5 and PD-1 monoclonal antibodies

**Figure 2:**
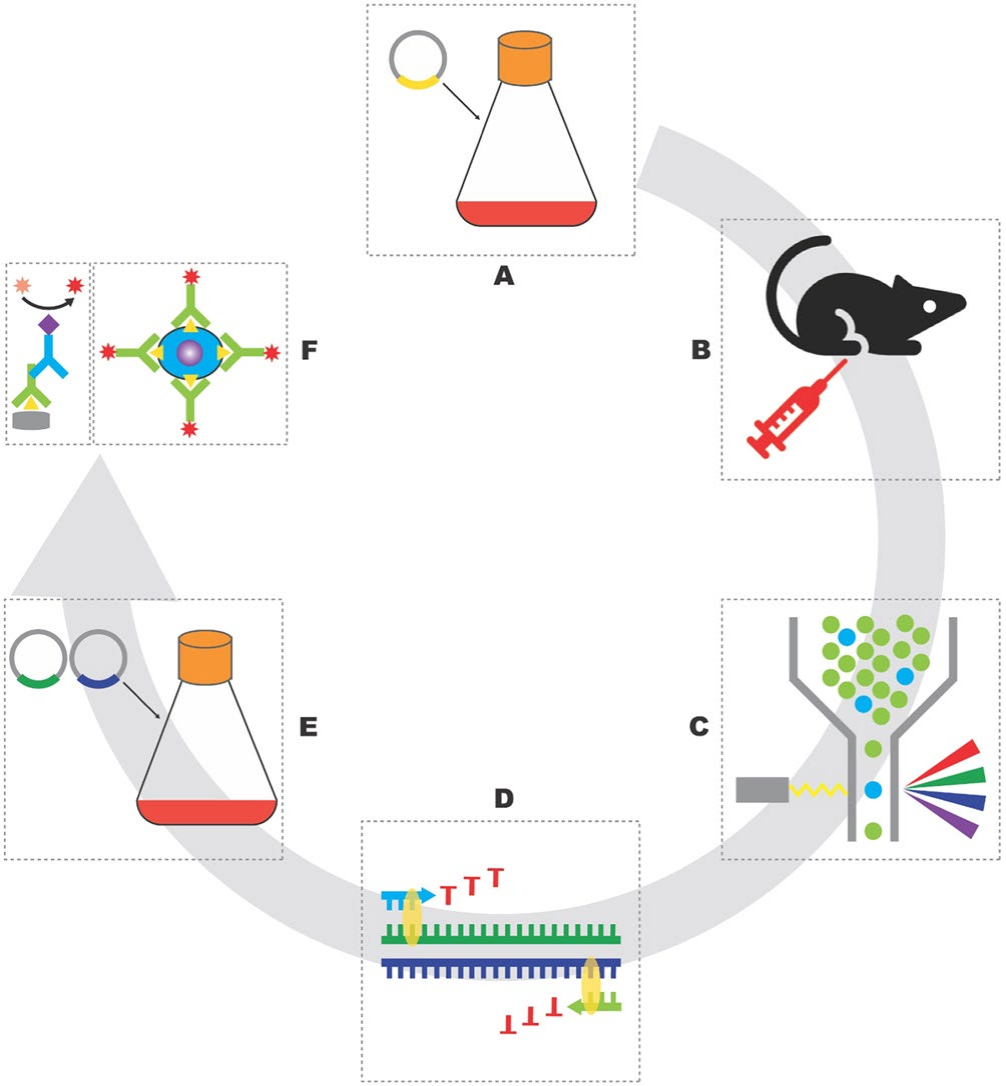
Flow chart to develop mouse anti-ferret CXCR5 and PD-1 monoclonal antibodies. Antigens were expressed by transiently transfection of Expi293 cells (**a**), C57BL/6 mice were intramuscularly immunized with purified antigens (**b**), antigen-specific B cells were single-cell-sorted by Aria III (**c**), the sequences of heavy and light chain variable domains of BCR were recovered by single cell PCR (**d**), candidate antibody clones were expressed by transiently transfection of Expi293 cells with heavy and light chain plasmids (**e**), purified antibodies were validated by ELISA and FACS (**f**). Illustration was drawn using Adobe Illustrator 2020.

Due to the low sequence conservation, we initiated the de novo development of anti-ferret PD1 and CXCR5 monoclonal antibodies for flow cytometric use (workflow in Fig. 2). The cDNA sequence of the ectodomains of ferret CXCR5 and PD-1 were identified using a NGS dataset (Wong et al. in press). These genes were synthesized and cloned into a mammalian expression vector containing human IgG1 Fc tag used for protein purification. Recombinant proteins were expressed by Expi293 cells and purified by protein A agarose. Next, we immunized C57BL/6 mice with recombinant ferret CXCR5 or PD-1 proteins. At day 21 post-immunization, we isolated draining lymph nodes from the mice, stained lymph node cell suspensions with a panel of antibodies as well as immunogen probes. The murine GC B cells binding to the fluorescent ferret CXCR5 or PD-1 probes were single-cell-sorted into 96-well PCR plates. The BCR sequences of sorted B cells were then recovered by single cell PCR with mouse IgG heavy or light chain primers^19^. The variable domains genes of heavy or kappa chains from clonally expanded families of B cells were synthesized and cloned into mammalian expression vectors containing mouse IgG1 or kappa chain constant domain gene. Antibodies were expressed in Expi293 and purified by protein G agarose.

### Validation of anti-ferret CXCR5 and PD-1 monoclonal antibodies by ELISA

**Figure 3:**
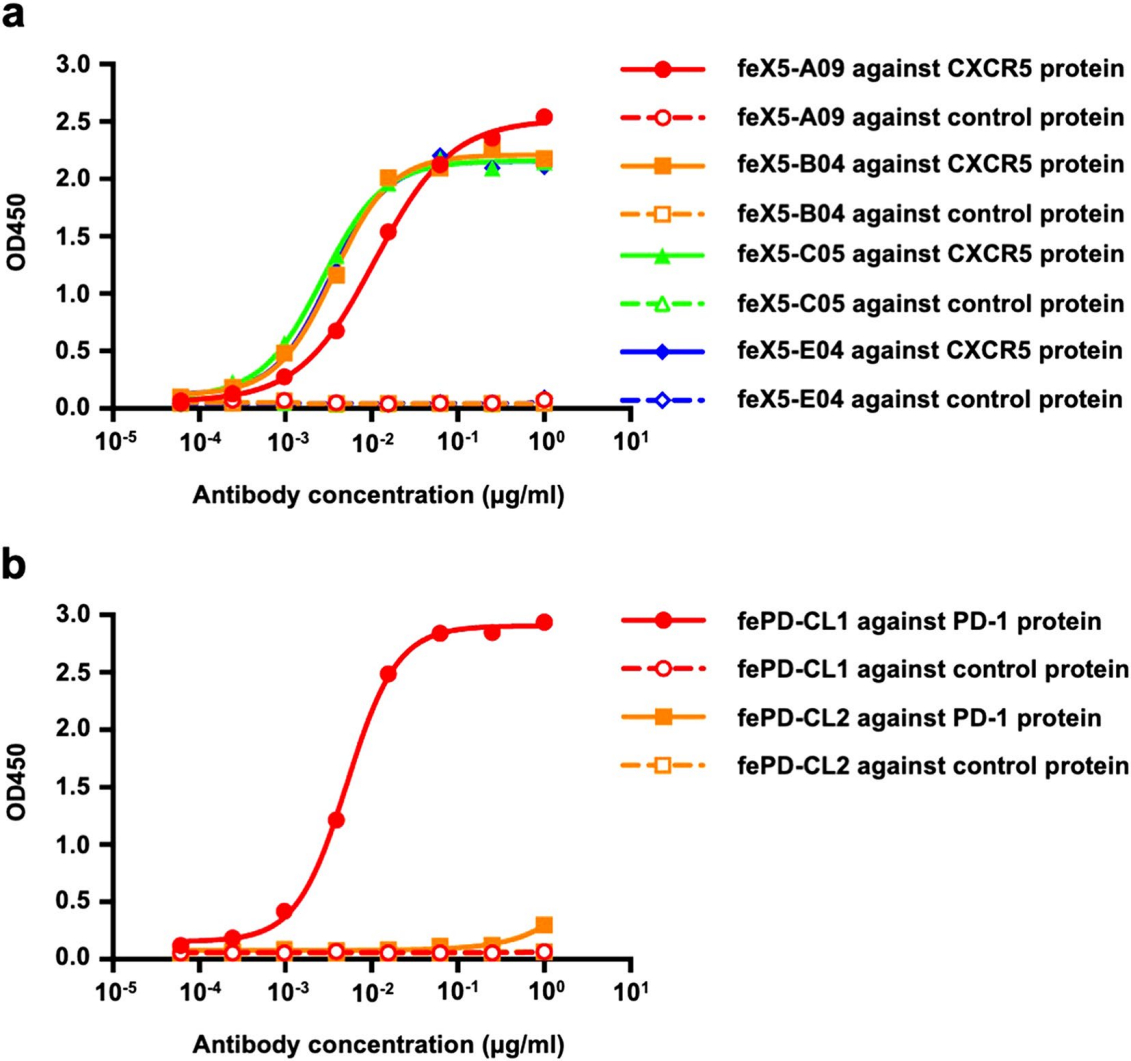
Binding activity of anti-CXCR5 and PD-1 antibodies with autologous and control antigens. (**a**) Binding activity of anti-ferret CXCR5 clone A09 (feX5-A09), B04 (feX5-B04), C05 (feX5-C05) and E04 (feX5-E04) against recombinant ferret CXCR5 protein or an irrelevant control protein with the same Fc tag. (**b**) Binding activity of anti-ferret PD-1 clone CL1 (fePD-CL1) and CL2 (fePD-CL2) against recombinant ferret PD-1 protein or an irrelevant control protein with the same Fc tag.

The binding specificity of the putative mouse anti-ferret CXCR5 and PD-1 antibodies was first assessed by ELISA. An irrelevant antigen with the same Fc tag as recombinant ferret CXCR5 or PD-1 proteins was used as a control. Anti-ferret CXCR5 clone A09 (feX5-A09), B04 (feX5-B04), C05 (feX5-C05) and E04 (feX5-E04) showed high binding activity with ferret CXCR5 proteins with an EC50 of 0.0109 μg/ml, 0.0036 μg/ml, 0.0027 μg/ml, 0.0033 μg/ml, respectively (Fig. 3a). Anti-ferret PD-1 clone CL1 (fePD-CL1) similarly displayed high binding activity with ferret PD-1 proteins with an EC50 of 0.0052 μg/ml while clone CL2 (fePD-CL2) showed no binding activity with ferret PD-1 proteins by ELISA (Fig. 3b). All antibodies showed no binding with control proteins, demonstrating that the antibodies were not targeted against Fc tag region of the recombinant proteins.

### Identification of Tfh cells in lymph node cells from influenza infected ferrets

**Figure 4:**
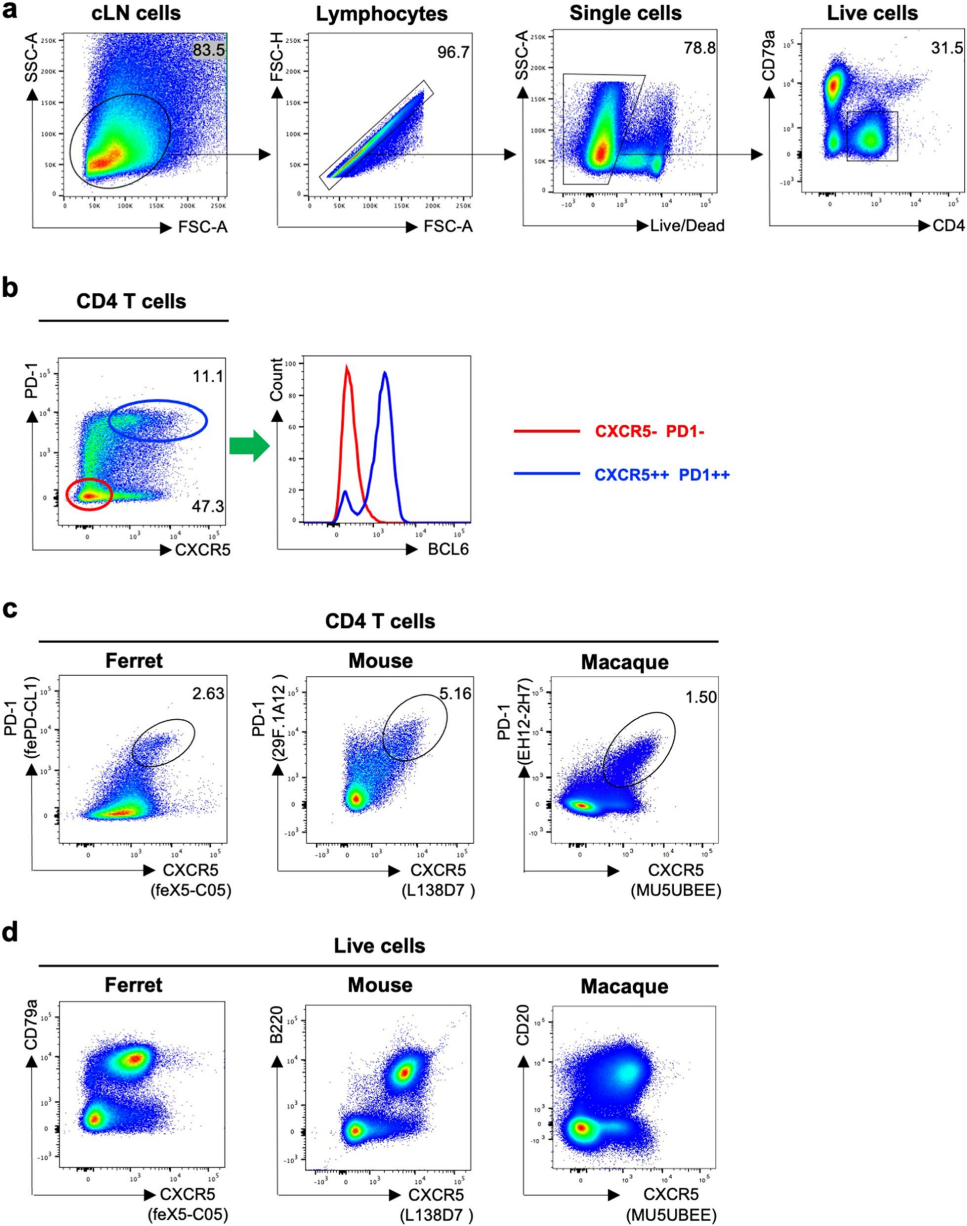
Identification of Tfh cells in lymph node cells from influenza infected ferrets. (**a**) Gating strategy to identify CD4 T cells in the ferret LN. Lymphocytes were identified by forward scatter area (FSC-A) and side-scatter area (SSC-A). Doublets were excluded by gating on single cells as determined by FSC-A versus FSC-H, live cells were identified by viability dye exclusion, CD4 T cells were identified as CD4+ CD79a−. For each step, the parental population is indicated above the plot. (**b**) Representative plots of ferret CD4 T cells stained with anti-BCL 6 (clone K112-91), anti-ferret CXCR5 (clone feX5-C05) and anti-ferret PD-1 (clone fePD-CL1) antibodies. The BCL6 expression of CXCR5++ PD-1++ CD4 T cells (Tfh, blue oval) were compared with CXCR5− PD-1− CD4 T cells (non-Tfh, red oval) cells. The parental population is indicated above the plot. (**c**) Representative plots of CD4 T cells from influenza-infected ferret, mouse and macaque LNs stained with species-appropriate CXCR5 and PD-1 antibodies (mouse, clones L138D7 and 29F.1A12 respectively; macaque, MU5UBEE and EH12.2H7 respectively). The parental population is indicated above the plot. (**d**) Representative plots of live lymphocytes from influenza-infected ferret, mouse and macaque LNs stained with their respective CXCR5 antibodies as well as CD79, B220 and CD19 antibodies. The parental population is indicated above the plot.

The ability of mAb feX5-C05 (anti-CXCR5) and fePD-CL1 (anti-PD1) to stain ferret lymphocytes was examined using flow cytometry. Single cell suspensions from the LN of influenza infected ferrets were stained with a panel consisting of anti-BCL6, CD4 and CD79a antibodies and anti-CXCR5 (feX5-C05) and anti-PD1 (fePD-CL1) conjugated to biotin and PE, respectively (gating in Fig. 4a). CXCR5++ PD-1++ CD4 T cells display elevated expression of BCL6 relative to non-Tfh cells (CD4+ CXCR5− PD−1−), consistent with a Tfh cell identity (Fig. 4b). Furthermore, the CXCR5 and PD-1 expression pattern of ferret CD4 T cells is similar to that of mouse and macaque CD4 T cells (Fig. 4c). In addition to CD4 T cells, CXCR5 is highly expressed by mouse and macaque B cells (Fig. 4d). Consistent with mouse and macaque data, we found that ferret CD79a+ B cells were also predominately CXCR5+ (Fig. 4d). Taken together, these data show that ferret Tfh cells are detected by our in-house developed anti-ferret CXCR5 and PD-1 antibodies or combination with a commercial cross-reactive anti-BCL6 antibody.

## Discussion

Protective antibody responses induced by vaccination play a central role in defense against virus infection^14^. Ferrets are a useful animal model for studying the pathogenicity and transmissibility of several human viruses and for pre‐clinical evaluation of the in vivo protective efficacy of vaccines. The help signal provided by Tfh cells is a critical factor which determines the magnitude and quality of antibody responses^20^. Identification of Tfh cells in ferrets could greatly assist providing an immunological rationale for the design of novel vaccines against the infection of influenza and other viruses, including SARS-CoV2. However, key immunological reagents, such as ferret reactive Tfh marker monoclonal antibodies, were lacking until this report.

In the present study, we developed ferret-specific CXCR5 and PD-1 monoclonal antibodies and identified Tfh cells in ferrets. Identification of Tfh cells by surface CXCR5 and PD-1 staining is of great utility if live Tfh cell staining is needed (such as for antigen induced activation or RNA studies) since intranuclear BCL6 staining involves cell fixation and permeabilization. In addition, CXCR5 could be a surrogate surface B cell marker if intracellular staining is not warranted or wanted since the currently available marker for ferret B cells (a commercial cross-reactive CD79a antibody) requires intracellular staining. Furthermore, anti-ferret PD-1 antibody can be useful for studies of non-TFH T cell activation or exhaustion in this animal model (as demonstrated in Fig. 4), thereby increasing the complexity and detail of immunophenotype studies.

Ferret Tfh responses remain largely unexplored, and basic questions, such as what the magnitude and quality of ferret Tfh responses are in the context of virus infection or vaccination, and how Tfh responses correlate with antibody responses following vaccination, remain to be answered. With these ferret-specific CXCR5 and PD-1 antibodies, these questions can be investigated, including longitudinal tracking of Tfh activity following virus infection. Results from ferret Tfh response studies can provide useful insights regarding how Tfh cells influence vaccine immunogenicity. These insights will assist the design and evaluation of novel Tfh-targeting vaccines against the infection of influenza and other viruses in ferrets, as they have for other animal models. Considering the current situation with coronavirus disease (COVID-19) pandemic, these reagents will be helpful in evaluation of the Tfh responses induced by COVID-19 vaccines in ferret model and accelerate the development of vaccines against SARS-CoV2 infection^9,10^.

**Figure 5:**
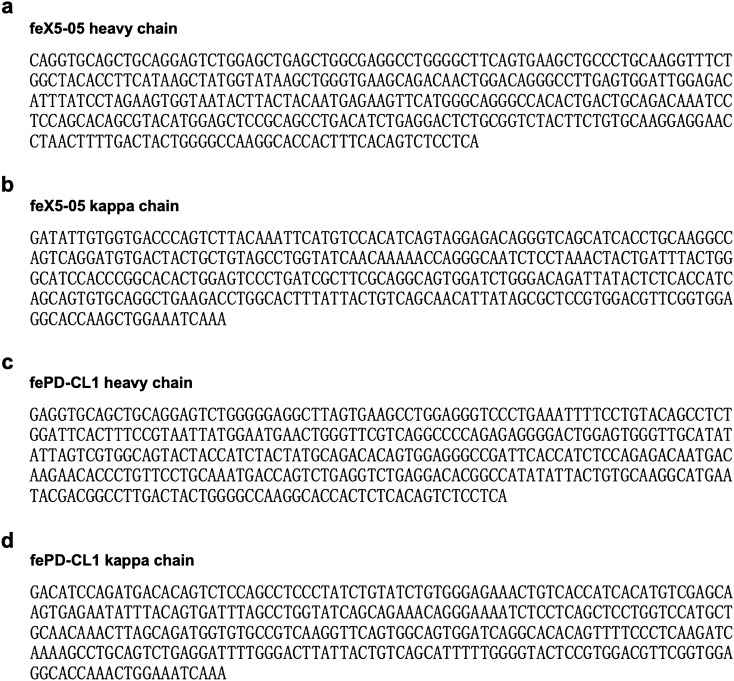
The nucleic acid sequences of variable domains of anti-ferret CXCR5 and PD-1 antibodies. (**a**) The nucleic acid sequences of variable domain of anti-ferret CXCR5 (clone feX5-C05) heavy chain. (**b**) The nucleic acid sequences of variable domain of anti-ferret CXCR5 (clone feX5-C05) kappa chain. (**c**) The nucleic acid sequences of variable domain of anti-ferret PD-1 (clone fePD-CL1) heavy chain. (**d**) The nucleic acid sequences of variable domain of anti-ferret PD-1 (clone fePD-CL1) kappa chain.

In summary, we developed ferret-specific CXCR5 and PD-1 monoclonal antibodies which were next used for detection of ferret Tfh cells. As these reagents identify surface-expressed antigens, they are compatible with live cell sorting and downstream RNA sequencing analysis, which can be challenging with antibodies requiring cell permeabilization and intracellular staining. The sequences of the heavy and light chain variable domains of anti-ferret CXCR5 and PD-1 antibodies are provided (Fig. 5) so that the field can use these reagents to advance the study of ferret Tfh. Recombinant antibodies can be expressed in a short time frame by transiently transfection of mammalian Expi293 cells. These ferret specific CXCR5 and PD-1 antibodies provide a starting point to allow in-depth study of the Tfh responses to viral infections, such as influenza and SARS-CoV2. Further reagents (such as anti-ferret CD154 monoclonal antibody) that are in critical need to identify antigen-specific Tfh are under development and will be made publicly available in the future.

## Materials and methods

### Animals and ethics

Mouse studies and related experimental procedures were approved by the University of Melbourne Animal Ethics Committee (#1914874) and were conducted in accordance with the Prevention of Cruelty to Animals Act (1986), the Australian National Health and Medical Research Council Code of Practice for the Care and Use of Animals for Scientific Purposes (1997) and the ARRIVE guidelines. Female C57BL/6 mice (6–8 weeks old) were immunized with 10 μg of recombinant ferret CXCR5 or PD-1 proteins with Addavax (1:1 ratio; InvivoGen) at both hind quadriceps using 29G needles. 21 days post vaccination, draining lymph nodes (inguinal lymph nodes (inLN) and iliac lymph nodes (ilLN)) were collected and mashed into single cell suspension using 70 μm strainer (Miltenyi Biotec) for staining.

Ferret specimens were collected from culled animals that were involved in experiments approved by The University of Melbourne Animal Ethics Committee (AEC 1714183) and were conducted in accordance with the Prevention of Cruelty to Animals Act (1986) and the Australian National Health and Medical Research Council Code of Practice for the Care and Use of Animals for Scientific Purposes (1997). Male ferrets were intranasally infected with between 1 × 10^4^ to 1 × 10^6^ TCID50 in 0.5 ml of A/Perth/265/2009. At day 14 post-infection, paratracheal lymph nodes from influenza-infected ferrets were removed, cut into small pieces and passing through a 70 μm strainer (Miltenyi Biotec). Single cell suspensions were then frozen in freezing media (90% fetal calf serum with 10% DMSO) and stored in liquid nitrogen.

As a comparator for pan-species expression of CXCR5 on T and B cell population, we assessed macaque Tfh and B cell phenotypes from lymph node samples. The macaque samples used in this were obtained from a macaque influenza vaccination trial and were processed as described previously^21^.

### Sequence analysis and recombinant protein generation

The nucleic acid sequences of ferret, human and mouse BCL6, CXCR5 and PD-1 were extracted from Ensembl website^22^ and Centre for Biotechnology Information (NCBI). Amino acid homology of ferret, human and mouse BCL6, CXCR5 and PD-1 were compared using Geneious.

The ectodomain of ferret CXCR5 and PD-1 were identified using the NGS dataset (Wong et al. manuscript submitted). The gene sequence of ectodomains of ferret CXCR5 and PD-1 were codon-optimized and synthesized (GeneArt) and cloned into mammalian expression vector containing human IgG1 Fc tag. Plasmids were extracted using NucleoBond Xtra Midi Plus plasmid DNA kit (MACHEREY-NAGEL). Recombinant ferret CXCR5 and PD-1 proteins were expressed by transient transfection of Expi293 (Thermo) suspension cultures with 2.7 μl ExpiFectamine (Thermo) and 1ug DNA/ml cell culture. At day 5 post-transfection, proteins in culture supernatant were purified by protein A agarose affinity chromatography and gel filtration.

### Sorting of ferret CXCR5 and PD-1 specific B cells

Recombinant ferret CXCR5 and PD-1 proteins were conjugated with APC or PE (Abcam) according to the manufacturer’s instructions. The resulting fluorescent proteins were designated as probes. Cell suspension of draining lymph nodes from ferret CXCR5 and PD-1 immunized mice were stained with probes and the following panel: live/dead Aqua (Thermo Fisher), CD45 APC-Cy7 (30-F11; BD), CD3 BV785 (145-2C11; BioLegend), F4/80 BV785 (BM8; BioLegend), Streptavidin BV785 (BD), B220 BV650 (RA3-6B2; BD), IgD PerCP-Cy5.5 (11-26c.2a, BD), CD38 PE-Cy7 (90; BioLegend), GL7 AF488 (GL7; BioLegend). Probe-binding GC B cells were single-cell-sorted to 96-well PCR plates a BD Aria III.

### RT-PCR

The BCR sequences of sorted ferret CXCR5 or PD-1 specific B cells were recovered as previously described^19^. Briefly, the mRNA of sorted single B cells was reversely transcribed into cDNA using SuperScript III reverse transcriptase (Thermo) and random hexamer primers (Bioline). The sequences of heavy and light chain variable domains were then amplified by nested PCR using HotStarTaq DNA polymerase (Qiagen) and mouse immunoglobulin heavy and light chain primers^19^. PCR products were sequenced in Macrogen.

### Antibody generation

The gene of heavy or kappa chain variable domain was synthesized (GeneArt) and cloned into mammalian expression vector containing mouse IgG1 or kappa chain constant domain. Heavy and kappa chain plasmids were extracted using NucleoBond Xtra Midi Plus plasmid DNA kit (MACHEREY-NAGEL). Antibodies were expressed by transient transfection of Expi293 (Thermo) suspension cultures with 2.7 μl ExpiFectamine (Thermo) and 1ug DNA (heavy: kappa = 1:1)/ml cell culture. At day 5 post-transfection, antibodies in culture supernatant were purified by protein G agarose affinity chromatography. For flow cytometric application, antibodies were conjugated to biotin or PE using biotin or PE conjugation kit (Abcam).

### ELISA

ELISAs were performed based on a modified protocol previously described^23^. 96-well MaxiSorp plates (Thermo) were coated with recombinant ferret CXCR5 or PD-1 or control proteins (1 μg/ml at 100 μl/well) overnight at 4 °C. After blocking with 2.5% BSA in PBS, anti-ferret CXCR5 or PD-1 antibodies at different dilutions (starting at 1 μg/ml, four-fold serial dilutions) were added and incubated for two hours at room temperature. Plates were washed with 0.05% Tween 20 in PBS prior to incubation with 1:5000 dilution of HRP-conjugated goat anti-mouse IgG (Sera-Care) for 1 h at room temperature. Plates were washed again and developed using 3,3′,5,5′-Tetramethylbenzidine (TMB) substrate (Sigma) and read at 450 nm using CLARIOstar microplate reader (BMG LABTECH).

### FACS

Lymph node cell suspensions from influenza infected ferret were stained with the following panel: live/dead Blue (Thermo Fisher), CD79a PerCP-Cy5.5 (HM47; BioLegend)^24^, CD8 AF700 (OKT8; Thermo)^24^, CD4 FITC (from CSIRO)^25^, BCL6 AF647 (K112-91; BD), BCL6 AF647 (IG191E/A8; BioLegend), CXCR5 BV421 (L138D7; BioLegend), CXCR5 BB515 (RF8B2; BD), CXCR5 PE (2G8, BD); CXCR5 Biotin (in-house), Streptavidin BV421 (BD), PD-1 BV786 (29F.1A12; BioLegend), PD-1 BV421 (EH12.2H7; BioLegend), PD-1 PE (in-house). For BCL6 staining, cells were fixed, permeabilized, and stained using the BD Transcription Factor Buffer kit (BD) according to the manufacturer’s instructions. Macaque LN suspensions were stained with Live/dead Aqua (Thermo Fisher), CD4 BV605 (L200; BD), CXCR5 PeCy7 (MU5UBEE, Thermo Fisher), and CD3 BUV737 (SP34-2, BD). All samples were acquired on a BD LSR Fortessa using BD FACS Diva and data was analyzed in FlowJo v10.
